# Supplementary material for: The Barriers, Challenges, and Strategies of COVID-19 (SARS-CoV-2) Vaccine Acceptance: A Concurrent Mixed-Method Study in Tehran City, Iran
Source: Vaccines (Basel). 2021 Oct 28;9(11):1248. doi: 10.3390/vaccines9111248 (PMC8620861; doi:10.3390/vaccines9111248)
Supplement: Supplementary file 1 [file vaccines-09-01248-s001.zip › vaccines-1393302-supplementary.pdf]

Supplementary Materials

Table S1. A summary of the implementation method and the results of some studies conducted in different parts of the world to determine the level of acceptance and skepticism concerning the COVID-19 vaccine.

Country	Effective Factors	Acceptance rate	The main question about the vaccine	Tool/Type of data collection	N	The time of conducting the study	The WHO-wise region	Complementary explanations
USA[1]	Factors affecting non-acceptance: Non-American race, low levels of education and income, being rural northeastern residents and Republican	Very likely (52%) Somewhat likely (27%) Not likely (15%) Definitely not (7%)	Question: "If a vaccine was available that would prevent coronavirus infection, how likely is it that you would receive the vaccine/shot" Responses: very likely, somewhat likely, not likely, definitely not	Researcher-made questionnaire / using Facebook, Twitter, academic posts, and social media platforms	1887	2020 January	Americas Region	
USA[2]	Factors affecting vaccine acceptance: Age of over 55, Indian-American race, being Asian or white	Agree/strongly agree (67%)	Question: "If a vaccine becomes available and is recommended for me, I would get it" strongly disagree/disagree/neuter =0 agree/strongly agree =1	An online questionnaire, using the Cloud Research platform	675	May 2020	Americas Region	The study was conducted in 10 US regions based on the DHS classification. In this study, no significant relationship was identified between the level of influenza vaccine coverage and the acceptance of the COVID-19 vaccine. The median risk perception of the study participants was 6 (IQR: 5-7).
Canada [3]	Factors affecting non-acceptance: Concerns about vaccine safety, Suspicion of political and/or economic forces forcing vaccination, the lack of knowledge about vaccines; Receiving anti-vaccine or confusing messages from the authorities, the lack of legal responsibility of vaccination companies, and the historical legacy of distrust of the medical industry by minority communities.	------	------	Reviewing Twitter using the keywords "Vaccine, Accepting Vaccine, and COVID-19"	Tweets: 605	2020 December	Americas Region	In this study, researchers reviewed approximately 3915 tweets in one week and selected 605 tweets that met the inclusion criteria of the study. This was a qualitative study and the rate of vaccine acceptance was not reported.

Indonesia[4]	In the scenario of 95% efficiency: being an employee of the healthcare system, high scores of perceived risk<br><br>In the 50% efficiency scenario: being an employee of the healthcare system	95% efficiency scenario: 93.3%<br>50% efficiency scenario: 67%	Question: Participants were asked to respond to the question of whether they would be vaccinated with a new COVID-19 vaccine for each scenario.<br><br>Response: “yes” or “no.”	Online questionnaire / WhatsApp<br>In this study, two scenarios were surveyed based on 50% and 95% efficacy of the vaccine	1068	March & April 2020	South-East Asia Region	In this study, 40% of the research participants believed that their risk of coronary heart disease was zero.<br>In this study, age, gender, level of education, occupation, religion, marital status, and income were not related to vaccination acceptance.
Malaysia[5]	Male gender, race, knowledge the infection of a friend, relative or family member, Perceived Risk, Belief in the severe complications of COVID-19	Yes, definitely: 48.2%<br>Yes, possibly: 16.3%<br>Yes, probably: 29.8%<br>Probably not: 3.3%<br>Definitely not: 2.4%	Question: If a vaccine against COVID-19 infection is available in the market, would you take it?<br><br>Response :five-point scale (‘Definitely not’ to ‘Yes, definitely’)	Online questionnaire / Using WhatsApp and Facebook to send the questionnaire link	1159	April 2020	South-East Asia Region,	In this study, 90% of the participants reported a university education.<br>Among the factors affecting acceptance, perceived barriers had the highest effect (OR=2.51). In this question, the participant was asked about the concerns that the adverse effects of COVID-19 vaccination may have and interfere with my normal activities<br>Response scale:<br>I agree/I disagree
Japan[6]	Factors affecting non-acceptance:<br>Female gender, age: 20 to 49 years, no underlying diseases	Very likely= 62%	Question: how likely they were to get vaccinated for COVID-19, once a vaccine is available to the public<br><br>Response: very unlikely, somewhat unlikely, somewhat likely, very likely, and unsure	Email invitation from an internet research company and using an online questionnaire	3000	January 2021	Western Pacific Region	A sampling of this study was performed on all regions of Japan
China [7]		The acceptance rate in the study participants in both phases: (n=791)<br>First phase: 91.91%,<br>Second phase: 88.6%, the acceptance rate for starters of the first phase: (n=2058)<br>91.3%	Question: "If a COVID-19 vaccine is successfully developed and approved for listing in the future, would you accept vaccination?"<br><br>Response: yes or no	An online questionnaire using the largest online study platform in China	791 participants in both phases and 2058 subjects starting the first phase	Phase one: March 2020<br>Phase two (follow-up): November and December 2020	Western Pacific Region	The first phase of this study was performed at the peak of the epidemic of the second phase in better conditions concerning COVID-19 prevalence.<br>The rate of immediate injection after vaccination was reduced from 52% to 23% due to vaccine safety concerns.
Australia [8]	Factors affecting doubt and rejection of vaccination:<br>Female gender, those living in marginalized areas, those with more	Decisive: 59%<br>Slight hesitation: 27%<br>Serious doubts: 7%<br>Definitive use: 6%		Online questionnaire	3000 adults aged over 18 years	December 2020	Western Pacific Region	The research findings suggested that vaccine suspicions, which make up a significant portion of the population, can be addressed by public health messages; how-

	populist views and higher religiosity							ever, it may be possible for a significant minority of individuals with strong beliefs. Other political measures are required to achieve adequate vaccination coverage to end the epidemic.
Italy [9]	Trust in scientific research, vaccine efficacy, age	%59	Question: participants were also asked to report their willingness to the vaccine for COVID-19 "if a vaccine was found"<br>Response: on a scale ranging from 1 (not likely at all) to 5 (absolutely likely).	(Norstat Italia, srl)<br><br>An online questionnaire using a professional platform in Italy (Norstat Italia, srl)	1004	2020	European Region	
France [10]	Factors affecting doubts about vaccine acceptance:<br>Female gender, young age, low level of education, non-adherence to past vaccinations, hypertension, awareness of acquaintances with COVID-19, presenting COVID-19 signs without medical approval, occupation, the manufacturing country of the vaccine,<br>Factors affecting the definitive acceptance of the vaccine:<br>Male gender, age, level of education, low understanding of the risk of COVID-19, non-adherence to past vaccinations, chronic diseases other than hypertension	Doubt in receiving the vaccine: 72%<br>Definite failure to receive the vaccine: 28%	Unspecified	The questionnaire was conducted online using the firm BVA platform.	2000	July 2020	European Region	In this study, all analyzes were performed based on the two consequences of definite rejection and delay in receiving the vaccine.<br><br>The acceptance rate of Chinese vaccines with a complication of 1 in 10000 is 27% and for European vaccines with a risk of a complication of 1 in 1000 was about 70%.<br><br>This study was performed in the age group of 18 to 64 years and 3 weeks after the first national quarantine.
Spain [11]	Factors affecting acceptance:<br>Being a nurse, other	Yes:77.5%	**Question**: Do you plan to get vaccinated against covid-19 when the vaccine is available<br>**Response**:<br>Yes or No	Online questionnaire using Twitter	731	January-March 2020	European Region	22.25% of the study participants stated that they would not use the COVID-19 vaccine. Reasons for disagreeing with vaccination varied, including:

								Ineffectiveness of the vaccine, the lack of immunity during vaccination, dangerous adverse effects of the vaccine; It is believed that vaccines are generally harmful or that COVID-19 does not exist. It is believed that these vaccines are not safe due to their rapid production. Having some chronic illness for which the vaccine is not recommended, the lack of evidence for COVID-19 vaccine; the belief that these vaccines may contain nanorobots that track subjects and control their thinking.
Saudi Arabia [12]	Factors affecting vaccine acceptance: marital status, perceived risk, and trust in the system	Yes:65%<br>No: 7%<br>Not sure= 28%	**Question:** If Vaccine Against Coronavirus is Available, I Will Take It<br><br>**Response**:<br>Yes or No or Not sure	An online questionnaire using Survey Monkey® platform and inviting participants by sending invitation links to WhatsApp, Twitter, and Telegram	992	2020	Eastern Mediterranean Region	-----
Kuwait [13]	With the reduction of vaccine efficacy from 90% to 50%, the acceptance rate significantly decreased.<br>It decreased from 32% with 90% efficiency to 9% with 50% efficiency.<br>Smoking, obesity, high-risk perception of the disease, and the effectiveness of the vaccine were other factors influencing acceptance	definitely will get vaccinated<br>(27.2%)<br>probably will get vaccinated<br>(25.9%)<br>definitely will not get vaccinated<br>25.6%<br>probably will not get vaccinated<br>21.3%	**Question:** If a vaccine for COVID-19 is approved by local and international health agencies, would you be willing to take the vaccine?”<br>**Response:**<br>definitely yes<br>probably yes<br>probably no<br>and<br>definitely no	Online questionnaire and the use of Twitter, Telegram, and WhatsApp.<br>Three scenarios of 50%, 75%, and 90% efficiency were evaluated in this study.	2368	August-September 2020	Eastern Mediterranean Region	Most study participants were more concerned about possible adverse effects (83.7%), the lack of information (82.3%), safety (71.8%), and questionable efficacy (69.9%). Individuals who wanted to receive the COVID-19 vaccine presented less concern than those who refused to receive the COVID-19 vaccine.
Jordan [14]	Factors affecting non-acceptance:<br>Female gender, educational level of below university, being single, high risk perception of COVID-19 risks	Yes: 36.8%<br>Not sure=26.4%<br>No: 36.8	**Question** willingness to be vaccinated against COVID-19 (once available)<br>**Response**:<br>Yes or No or not sure	Online questionnaire and using Google Forms	1144	2020 October	Eastern Mediterranean Region	Sampling was conducted from 17 provinces of Jordan.<br>In this study, the major barriers to vaccine rejection were reported in 4 subcategories: concerns about the vaccine, the need for more information, attitude, and distrust. In each of these 4 subgroups, respectively,

								concerns about the adverse effects of the vaccine, expressing ignorance of the different dimensions of the vaccine, prioritizing individuals at higher risk for vaccine injection and testing, and not adhering to the vaccine due to political pressures to approach vaccines were the most common challenge addressed by the research participants.
Congo [15]	Factors affecting vaccine acceptance: Medium and high income, not working in the medical section, being suspected of having COVID-19.	55%/9	**Question**: Would you consent to receive a COVID-19 vaccine if it becomes available in our country? Response: yes or no	An online questionnaire using the Covidien platform; International Citizen Project COVID-19 (ICPCOVID)	4160	August-September 2020	African Region	Among 1821 participants who refused to receive the COVID-19 vaccine, the majority (n=1104, 60.6%) stated that they did not trust the vaccine. 262(14.4%) subjects believed that the vaccine was made to kill subjects in Africa and 108(5.9%) believed that the vaccine was made to sterilize individuals.

Refrences

1. Khubchandani J, Sharma S, Price JH, Wiblishauser MJ, Sharma M, Webb FJ. **COVID-19 vaccination hesitancy in the United States: a rapid national assessment**. *J Community Health* 2021; **46**(2): 270-7.
2. Malik AA, McFadden SM, Elharake J, Omer SB. **Determinants of COVID-19 vaccine acceptance in the US**. *EClinicalMedicine* 2020; **26**: 100495.
3. Griffith J, Marani H, Monkman H. **COVID-19 vaccine hesitancy in Canada: a content analysis of Tweets using the Theoretical Domains Framework**. *J Med Internet Res* 2021.
4. Harapan H, Wagner AL, Yufika A, et al. **Acceptance of a COVID-19 vaccine in southeast Asia: A cross-sectional study in Indonesia**. *Front Public Health* 2020; **8**.
5. Wong LP, Alias H, Wong P-F, Lee HY, AbuBakar S. **The use of the health belief model to assess predictors of intent to receive the COVID-19 vaccine and willingness to pay**. *Hum Vaccin Immunother* 2020; **16**(9): 2204-14.
6. Machida M, Nakamura I, Kojima T, et al. **Acceptance of a COVID-19 Vaccine in Japan during the COVID-19 Pandemic**. *Vaccines* 2021; **9**(3): 210.
7. Wang J, Lu X, Lai X, et al. **The Changing Acceptance of COVID-19 Vaccination in Different Epidemic Phases in China: A Longitudinal Study**. *Vaccines (Basel)* 2021; **9**(3).
8. Edwards B, Biddle N, Gray M, Sollis K. **COVID-19 vaccine hesitancy and resistance: Correlates in a nationally representative longitudinal survey of the Australian population**. *PLoS One* 2021; **16**(3): e0248892.
9. Palamenghi L, Barello S, Boccia S, Graffigna G. **Mistrust in biomedical research and vaccine hesitancy: the forefront challenge in the battle against COVID-19 in Italy**. *Eur J Epidemiol* 2020; **35**(8): 785-8.
10. Schwarzinger M, Watson V, Arwidson P, Alla F, Luchini S. **COVID-19 vaccine hesitancy in a representative working-age population in France: a survey experiment based on vaccine characteristics**. *Lancet Public Health* 2021; **6**(4): 210-21.
11. Eguia H, Vinciarelli F, Bosque-Prous M, Kristensen T, Saigí-Rubió F. **Spain's Hesitation at the Gates of a COVID-19 Vaccine**. *Vaccines (Basel)* 2021; **9**(2).
12. Al-Mohaithef M, Padhi BK. **Determinants of COVID-19 vaccine acceptance in Saudi Arabia: a web-based national survey**. *J Multidiscip Healthc* 2020; **13**: 1657.
13. Alqudeimat Y, Alenezi D, AlHajri B, et al. **Acceptance of a COVID-19 Vaccine and its Related Determinants among the General Adult Population in Kuwait**. *Med Princ Pract* 2021.
14. Al-Qerem WA, Jarab AS. **COVID-19 Vaccination Acceptance and Its Associated Factors Among a Middle Eastern Population**. *Front Public Health* 2021; **9**: 632914.
15. Ditekemena JD, Nkamba DM, Mutwadi A, et al. **COVID-19 Vaccine Acceptance in the Democratic Republic of Congo: A Cross-Sectional Survey**. *Vaccines (Basel)* 2021; **9**(2).
